# Supplementary material for: Conditional chemoconnectomics (cCCTomics) as a strategy for efficient and conditional targeting of chemical transmission
Source: eLife. 2024 Apr 30;12:RP91927. doi: 10.7554/eLife.91927 (PMC11060718; doi:10.7554/eLife.91927)
Supplement: Figure 3—source data 3. [file elife-91927-fig3-data3.zip › Figure 3 source data3 part 1.zip!/Figure 3-Source data 3-part1/1071-1-CRISPResso_Report_osfbjvga.zip!/CRISPResso_on_1071_4/9.Alleles_frequency_table_around_sgRNA_gaccgtctcaccgtcaagat.pdf]

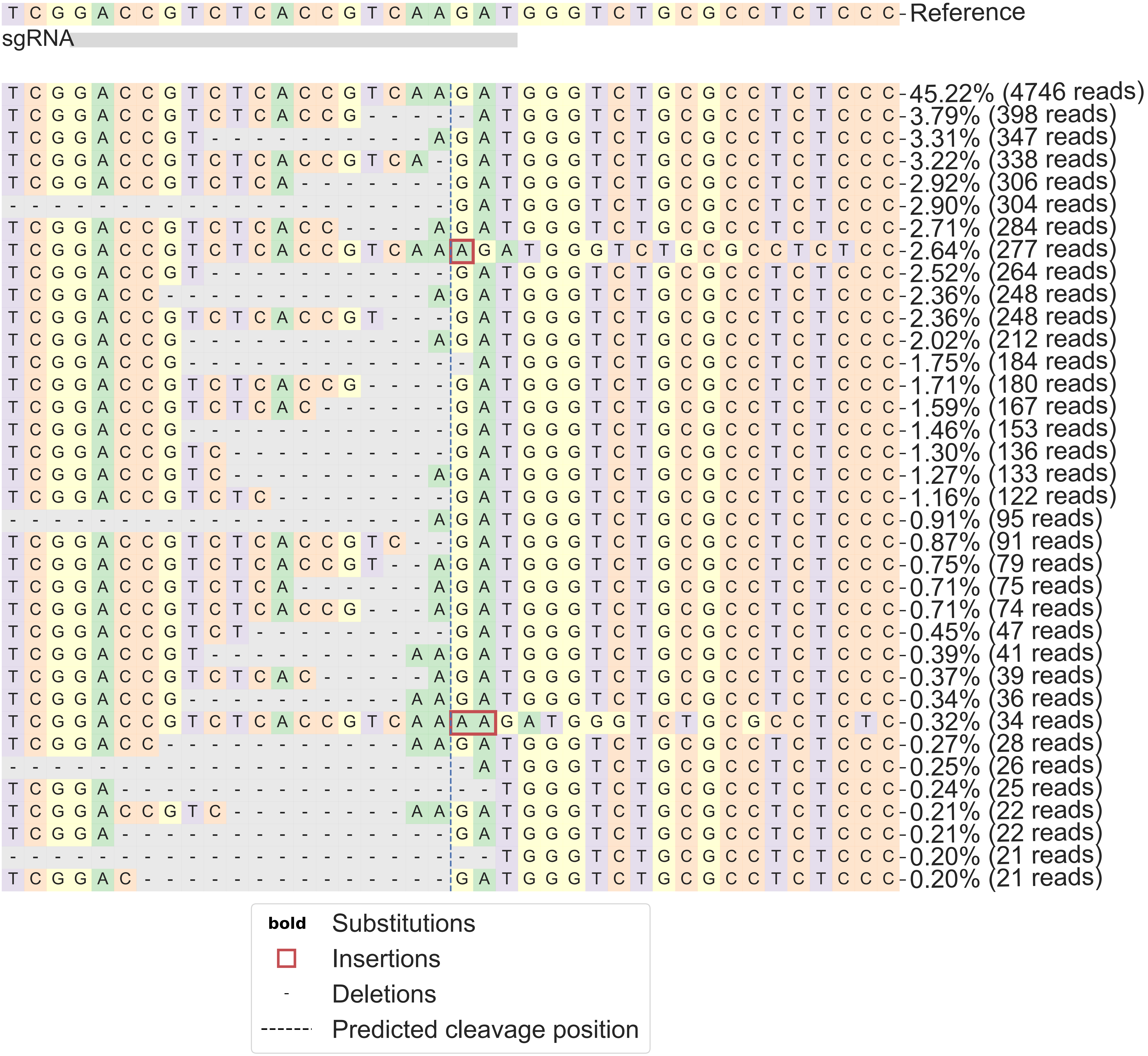
TCGGACCGTCTCACCGTCAAGATGGGTCTGCGCCTCTCCC Reference
sgRNA
45.22% (4746 reads)
3.79% (398 reads)
3.31% (347 reads)
3.22% (338 reads)
2.92% (306 reads)
2.90% (304 reads)
2.71% (284 reads)
2.64% (277 reads)
2.52% (264 reads)
2.36% (248 reads)
2.36% (248 reads)
2.02% (212 reads)
1.75% (184 reads)
1.71% (180 reads)
1.59% (167 reads)
1.46% (153 reads)
1.30% (136 reads)
1.27% (133 reads)
1.16% (122 reads)
0.91% (95 reads)
0.87% (91 reads)
0.75% (79 reads)
0.71% (75 reads)
0.71% (74 reads)
0.45% (47 reads)
0.39% (41 reads)
0.37% (39 reads)
0.34% (36 reads)
0.32% (34 reads)
0.27% (28 reads)
0.25% (26 reads)
0.24% (25 reads)
0.21% (22 reads)
0.21% (22 reads)
0.20% (21 reads)
0.20% (21 reads)
bold Substitutions
Insertions
Deletions
Predicted cleavage position
